# Recent clinical and mechanistic insights into vitiligo offer new treatment options for cell-specific autoimmunity

**DOI:** 10.1172/JCI185785

**Published:** 2025-01-16

**Authors:** Khaled Ezzedine, Rim Tannous, Todd F. Pearson, John E. Harris

**Affiliations:** 1 Department of Dermatology, Hôpital Henri Mondor, Université Paris-Est Créteil Val de Marne-Université Paris, Paris, France.; 2 EpidermE, Université Paris-Est Créteil (UPEC), Créteil, France.; 3 Department of Dermatology, UMass Chan Medical School, Worcester, Massachusetts, USA.

## Abstract

Vitiligo is an autoimmune disease that has been recognized, stigmatized, and treated for millennia. Recent translational research has revealed key mechanisms of disease, including cellular stress, innate immune activation, T cell–mediated elimination of melanocytes from the skin resulting in clinically apparent white spots, as well as stem cell regeneration that reverses established lesions. Many of these pathways have been targeted therapeutically, leading to the first FDA-approved medication to reverse the disease, with many more in clinical trials. Despite these impressive advances, many questions remain, which will be answered through integration of additional basic, translational, and clinical research studies. This vitiligo revolution has led to great excitement for individuals with vitiligo, those who know them, and the dermatologists who care for their patients. But just as importantly, these advances have great potential to shed light on autoimmune diseases that are more difficult to study, possibly leading to treatment advances that could not be achieved otherwise.

## Introduction

Vitiligo is an autoimmune disease of the skin characterized by the selective loss of melanocytes. This results in well-defined, chalky white spots that can appear on any part of the body, but occur most frequently on the face, hands, and genitals (1). Vitiligo is usually a clinical diagnosis, aided by Wood’s lamp examination to enhance the depigmented lesions. While rarely needed, skin biopsy is characterized by complete loss of melanocytes in the epidermis in established lesions, as well as T cells infiltrating the epidermis in close proximity to melanocytes in lesions that are actively spreading (2, 3).

In most cases, vitiligo does not directly cause systemic illness; however, it is associated with social and cultural stigma, with origins tracing back to ancient times. In various cultures, the disease was perceived as a divine punishment, a manifestation of demonic influence, or mistaken for leprosy (4). Ancient Indian medical texts indicated that people with vitiligo and their relatives could not marry, while Buddhist writings excluded those with vitiligo from ordainment. Therefore, social stigma is deeply ingrained for those with vitiligo, who often experience psychosocial challenges that differ depending upon the affected individual’s ethnic and cultural background (5). Treatments throughout history ranged from animal urine or dung applied topically and cobra bones taken orally during the Iron Age, to the use of arsenic, sulfuric acid, and other toxins within the past 150 years. The most notable ancient remedy was outlined in the Atharvaveda written in 1400 BC, which directed patients to chew on seeds from the *Psoralea* *corylifolia* plant and then sit in the midday sun. This approach was rediscovered in the 1950s when patients were treated topically or orally with purified psoralen (a compound present in the seeds) plus ultraviolet A light (PUVA therapy) (6).

Because of its significant social stigma, the impact on the quality of life (QoL) of patients has been studied extensively, both to help manage patient psychological health, as well as in the context of clinical trials to measure improvement with therapy. The extent and location of disease each impact QoL, with greater disease burden and more visible location associated with poor outlook (7, 8), and several tools have been developed to measure these factors (9, 10). Skin phototype and cultural background also impact the patient’s perception of the disease; for instance, darker skin increases the contrast/visibility of lesions, and in south Asian culture, individuals with vitiligo experience greater stigma due to history and culture (11). A general screening in a world-wide study using tools such as PHQ-9 has shown that anxiety (28.8%) and depression (24.5%) are common in individuals with vitiligo (12), at rates much higher than the general population.

### Vitiligo as a “model” autoimmune disease.

In his 2009 Banting Lecture, renowned diabetes researcher George S. Eisenbarth noted the similarity between the histopathology seen in the pancreas of individuals with type 1 diabetes (T1D) and the patchy destruction of melanocytes in vitiligo (13). This highlights how vitiligo shares mechanistic similarities with many other autoimmune diseases. However, vitiligo possesses important characteristics that make it an ideal candidate as a model autoimmune disease. Chief among these are the ease of access to the anatomical site of the autoimmune attack, presence of affected and unaffected tissue in the same individual, and a source of melanocyte stem cells that allow for repigmentation studies to assess disease reversal as well as autoimmune recurrence. To highlight the value of vitiligo as a model of cell-specific autoimmune disease, areas of congruence between vitiligo and other autoimmune diseases will be noted throughout this Review. Finally, vitiligo not only affords opportunities to generalize autoimmune mechanisms and treatment, but it also provides insight into melanoma immunotherapy as a prototypic antitumor response.

## Clinical presentation and differential diagnosis

**Figure 1:**
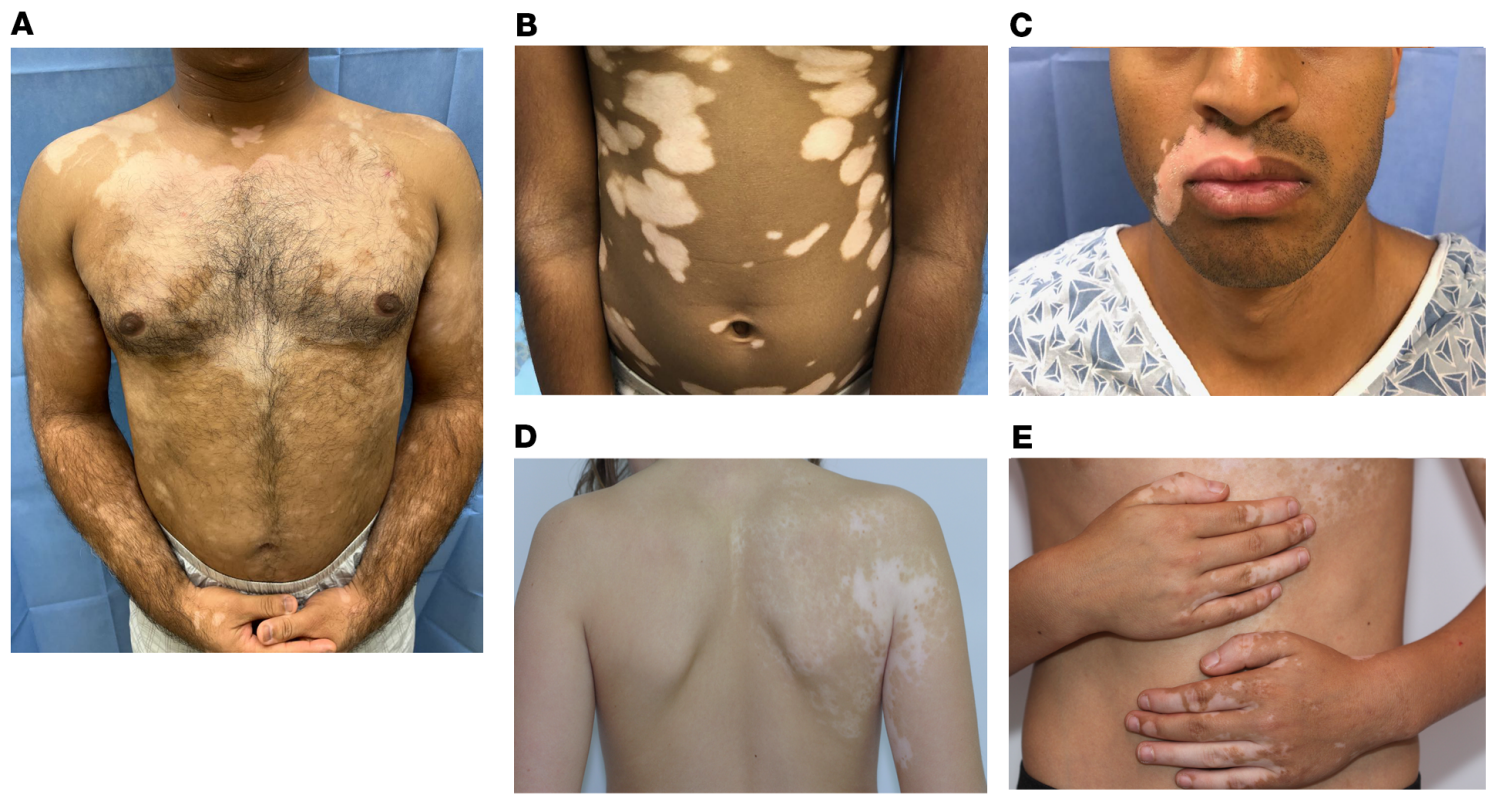
Clinical presentations of vitiligo. (**A** and **B**) Individuals with nonsegmental vitiligo, displaying characteristic symmetrical lesions on the body. Note normal body hair pigmentation in **A**. (**C** and **D**) Segmental vitiligo, with asymmetric lesions limited by the midline. Note depigmented lesional hairs in **C**. (**E**) Mixed vitiligo, characterized by segmental lesions that stop at the midline on the left anterior trunk as well as symmetric nonsegmental lesions on the hands. Photos are shown with patient consent.

Vitiligo most commonly presents with symmetrical lesions in multiple areas of the body (Figure 1, A and B). However, a variant of the disease called segmental vitiligo is characterized by a unilateral distribution of lesions that do not cross the midline (Figure 1, C and D). The term “nonsegmental vitiligo” is frequently used to describe the more common symmetrical form. The rare coexistence of segmental and nonsegmental vitiligo is called “mixed vitiligo,” which is characterized by at least one lesion limited by the midline and additional lesions that are symmetrical (Figure 1E). Multisegmental vitiligo describes a patient with segmental vitiligo and more than one lesion that is each limited by the midline. Focal vitiligo applies to localized lesions that may progress to either segmental or nonsegmental vitiligo.

**Figure 2:**
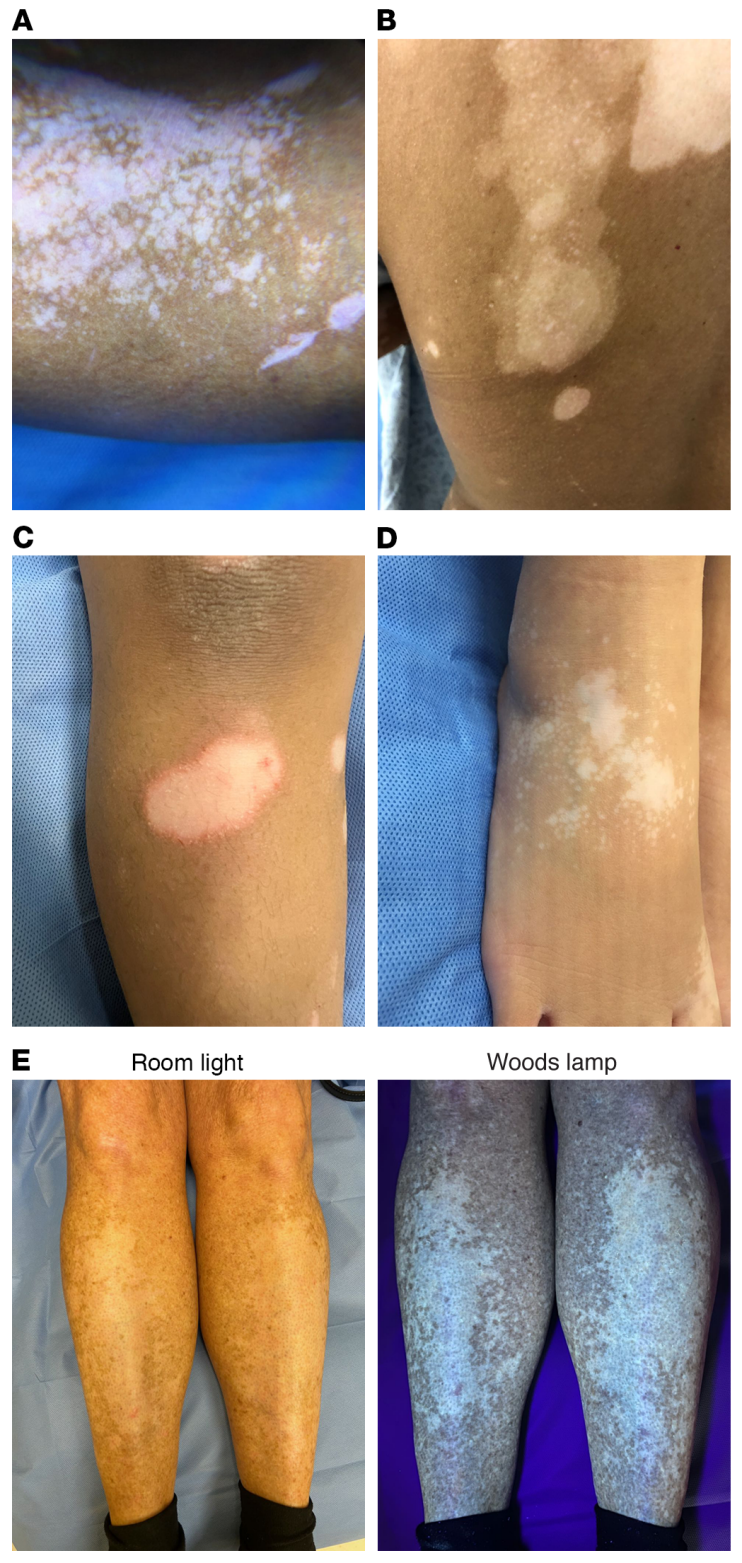
Clinical signs of active vitiligo lesions. (**A**) Koebner phenomenon illustrated by focal, linear depigmentation at a site of previous injury, indicated by arrow. (**B**) Trichrome lesions on the trunk, characterized by 3 colors including normal skin, depigmented skin, and intervening hypopigmentation. (**C**) Inflammatory vitiligo on the lower leg, with erythema and scale present at the lesional border, but sparing the center. (**D**) Confetti vitiligo on the foot, represented by clusters of small depigmented macules. (**E**) Lesions in fair skin can be difficult to appreciate by room light, while lesions fluoresce under illumination by Wood’s lamp, making examination more accurate. Photos are shown with patient consent.

In addition, individual lesions are characterized as either stable, without change in size or shape for 6 or more months, or actively spreading, when lesions can increase in size rapidly. Clinical signs of active lesions include the Koebner phenomenon (new lesions in areas of skin trauma, Figure 2A), trichrome lesions (3 colors marked by normal, depigmented, and intervening hypopigmented skin; Figure 2B), inflammatory lesions (erythema and scale at the lesion border, Figure 2C), and confetti-like depigmentation (clustered macules of depigmentation, Figure 2D) (14–16). Thus, clinical evaluation should define disease subtype, extent, and activity, as these factors will determine the individual treatment approach (17). Wood’s lamp examination, or illumination with UVA light or “black light” with room lights off, enhances visibility of lesions, particularly in those with lighter skin (Figure 2E). Importantly, disease activity should be considered when collecting samples for translational research to investigate disease pathogenesis, as more common stable lesions lack significant immune infiltration.

The differential diagnosis of vitiligo includes primarily hypopigmented, rather than depigmented, conditions such as nevus depigmentosus, pityriasis alba, tinea versicolor, idiopathic guttate hypomelanosis, hypopigmented mycosis fungoides, and other disorders (18). These are often easily distinguished from vitiligo using a Wood’s lamp examination, as they are hypopigmented rather than depigmented and therefore do not fluoresce under UVA light. Because vitiligo can accompany spontaneous regression of melanoma, a total skin exam should be performed to rule out an occult melanoma (19).

## Epidemiology

In a recent systematic review and modeling study, the worldwide lifetime prevalence of vitiligo diagnosed by a physician was estimated to be 0.36%, whereas undiagnosed but self-reported disease revealed an additional prevalence of 0.55%, thus affecting up to 0.91% (73 million people) worldwide (20). Vitiligo occurs in two primary age-of-onset categories: one-third of cases with early onset (mean 10.3 years) and two-thirds with later onset (mean 34.0 years) (21). A pediatric to adult shift in vitiligo onset was noted over a period of 62 years; the mean age of onset shifted from 14.7 ± 9.3 years in 1951 to 31.8 ± 20.2 years in 2013, a phenomenon that was consistent in both North America and Europe. This remarkable change might be explained by new environmental exposures of adults to vitiligo-inducing chemicals, the use of sunscreens reducing UV exposure that controls disease, an altered gut microbiome from a variety of factors, or other causes (22). Indeed, several chemicals have been reported to induce vitiligo, such as those present in permanent hair dyes, cleaning products, cosmetics, and other household products. In addition, the methylphenidate skin patch was reported to induce vitiligo in over 50 patients (23).

### Genetics of vitiligo.

Genome-wide associations studies (GWAS) in vitiligo identified close to 60 risk alleles that contribute to induction of disease (24). Over 90% of the presumed genes affected by these SNPs are central to immune responses, supporting a clear role of autoimmunity as a driver of pathogenesis. The most notable are *HLA* genes, which encode the MHC molecules responsible for antigen presentation to T cells, including HLA-A*02:01 and HLA-DQB1*02:02 (21, 25). Other GWAS-identified risk alleles in vitiligo implicate PTPN22, IL-2RA, CCR3, FoxP3, and others (26). Interestingly, many of these vitiligo-associated risk alleles have been identified in other autoimmune diseases, which might explain another important feature seen in many individuals with vitiligo and their family members: polyautoimmunity, characterized by the presence of multiple autoimmune diseases occurring in a single patient (27). This genetic overlap substantiates vitiligo as a model of diseases with shared genetic predisposition.

Despite clear association of some SNPs with immune genes, the majority are located in noncoding DNA (26), making it difficult to determine their role in pathogenesis, or even to define which gene is regulated by the SNP. Recent data reveal that 3D chromatin structures position regulatory regions of DNA close to regulated genes, even when found quite far from the gene in linear space (up to 70 kb) (28). Thus, SNPs frequently do not regulate the nearest gene, making functional genomic studies more difficult. These studies will require mapping of 3D structures in relevant cell types to better indicate how to uncover the mechanism of genetic predisposition toward vitiligo and related diseases.

## Vitiligo pathogenesis

### Vitiligo is an autoimmune disease.

For many years, the pathogenesis of vitiligo was debated, with different hypotheses proposed to explain the cause of melanocyte loss, including melanocyte degeneration through the cellular stress response, autoimmunity, genetics, and even neuropathic mechanisms (6, 29). However, basic, translational, and clinical studies indicate that both immune-mediated mechanisms and melanocyte stress likely contribute synergistically, with underlying genetic influences promoting each. Early advances in the immunology of melanoma provided research tools and causative insight into vitiligo, as successful antitumor responses, including responses to newer immunotherapeutic drugs, encompass similar pathways. Tools include mouse models of melanocyte immunity, isolation of T cell clones and their T cell receptors (TCRs), multimer staining of T cell specificity by flow cytometry, and genetic insights (6).

### Melanocyte biology.

Early studies of melanocytes from patients with vitiligo revealed that they were abnormal when compared with those from healthy individuals. They were more difficult to culture and demonstrated elevated levels of cellular stress, including activation of the unfolded protein response and presence of reactive oxygen species (30–32). Furthermore, certain chemical exposures were found to induce and exacerbate vitiligo in patients, such as the presence of monobenzone in gloves used by factory workers in the early 1900s (33) and more recently the use of permanent hair dyes or skin-lightening cosmetics (34). These chemicals, most of which are phenols, resemble the structure of the amino acid tyrosine, a building block of melanin, and thus interfere with the energy-demanding process of melanogenesis, resulting in increased cellular stress and release of damage-associated molecular patterns such as heat shock proteins like HSP70 (35–37) that activate cytokine release and inflammation (38). This likely leads to activation of innate immunity and ultimately adaptive responses that target melanocytes for destruction (39). These examples highlight the fact that melanocytes are not simply bystanders in vitiligo progression, but play an active role in their own destruction. Parallel observations about target cell stress have been made in other autoimmune diseases, such as T1D, multiple sclerosis (MS), and autoimmune thyroiditis (40).

### The role of the exposome.

Despite strong genetic associations with vitiligo and other autoimmune diseases, these do not account for the entirety of disease susceptibility. For example, the concordance rate for vitiligo in monozygotic twins is approximately 23% (41). While this could be due in part to stochastic influences on autoimmunity, such as chance recombination events that create the TCRs and B cell receptors and thymic selection of T cells, the environment is likely a contributor to initiation of autoimmunity as well. Thus, a role for the exposome (42), that is, the sum total of all environmental exposures, is likely implicated. The chemical exposures discussed above represent a well-defined environmental cause of autoimmunity in vitiligo, but other components of the exposome have also been described. All relevant components of the exposome deserve further investigation to better understand individual mechanisms that promote autoimmunity through exposures, some of which we outline below.

The microbiome has been hypothesized to influence autoimmunity in a variety of organ systems. Gut dysbiosis has been described in multiple diseases, including MS, systemic lupus erythematosus, T1D, and rheumatoid arthritis (43). Likewise, viral infections have been proposed as important infections that promote autoimmunity in a number of diseases (44), including the identified association between EBV and MS (45). Little work has been done to define the role of microorganisms in driving vitiligo, but several studies indicate a potential role. One revealed the key role of the gut microbiome in responsiveness of patients with melanoma to immune checkpoint inhibitor therapy (46), a strong correlate to vitiligo. Another study in a mouse model of vitiligo reported disease modulation following treatment with oral antibiotics, suggesting that microorganisms in the gut might influence autoimmune attack in the skin (47). However, this study did not identify the mechanism of this effect, or completely rule out a direct antiinflammatory effect of the antibiotics on vitiligo autoimmunity.

Since sunlight clearly improves vitiligo, it is another likely source of environmental influence on disease. Those in warmer climates and greater sun exposure may have a lower incidence or severity of disease; however, defining disease incidence is challenging because not all cases are recognized or reported. Anecdotal reports abound in our clinics (Hôpital Henri Mondor and UMass Chan Medical School) from patients with vitiligo who are convinced that moving from an area of high sunlight exposure to low exposure correlated with new onset of disease.

Emotional and psychological stressors are another component of the exposome implicated in vitiligo. Patients with vitiligo reportedly have a higher amount of emotional stress compared with those without vitiligo (48), and anecdotal reports of stress from patients correlating with worsening disease are prevalent in our clinics (Hôpital Henri Mondor and UMass Chan Medical School). While this association does not prove causality, studies in a mouse model of vitiligo indicate that vitiligo is exacerbated when mice are placed under conditions of chronic stress, which was attributed to glucocorticoid signaling in macrophages (49). Another hypothesis for how stress triggers autoimmunity is through changes in the gut microbiome and defects in the intestinal barrier (50). In addition, the geographical move described above could promote vitiligo through stress. Stress is difficult to quantify, and thus reports of direct association between vitiligo and stress are scarce. Future studies to quantify this association and highlight specific stressors will be important for vitiligo and related autoimmune diseases.

### T cell–mediated autoreactivity.

The best characterized component of disease pathogenesis in vitiligo is the role of CD8^+^ T cells in the targeted destruction of melanocytes (51–53). These studies were inspired by early observations in melanoma immune responses, including the presence of tumor-infiltrating lymphocytes that were CD8^+^, IFN-γ producing, and melanocyte antigen specific (54). These cells targeted and killed melanocytes in vitro, and even shrunk tumors in vivo after removal, expansion, and activation (55, 56). A common and even expected side effect of this process through effective antitumor responses was depigmentation of the skin, reminiscent of vitiligo (57). In patients with vitiligo, cells with similar specificities and effector mechanisms were identified first in the blood (52, 58), and later the skin (59).

Mouse models developed to investigate mechanisms of melanoma indicated a clear role for melanocyte-reactive CD8^+^ T cell clones, as well as their reliance on IFN-γ and cytotoxic effector mechanisms. One model in particular, based on inoculation of B16 melanoma cells into the skin followed by adoptive transfer of TCR-transgenic CD8^+^ T cells specific for premelanosome protein (PMEL) (60), was later adapted to create a simpler vitiligo model by eliminating the B16 inoculation and using a host with epidermal pigmentation for the transfer (61). T cells were activated either by infection with vaccinia virus expressing PMEL or cotransfer with dendritic cells pulsed with PMEL peptide antigen (62, 63). Additional models exist, including priming with melanoma followed by excision and Treg depletion, or exposure to high-dose monobenzone (64, 65), and a number of animals have been reported to develop vitiligo spontaneously, including chickens, pigs, dogs, horses, and others (66).

Combined studies using vitiligo mouse models as well as isolation of cells and tissues from patients with vitiligo confirmed a key role for IFN-γ–producing CD8^+^ T cells in vitiligo pathogenesis (52, 59, 61, 67). Further studies revealed that IFN-γ induced the expression of CXCL9 and CXCL10 chemokines by keratinocytes and fibroblasts to recruit additional T cells from the blood into the skin, directing their localization into the dermis and then up to the epidermis where melanocytes are targeted (68–71). This promotes a positive feedback loop that drives the progression of vitiligo. One study reported that fibroblasts located in different regions of the body responded differently to IFN-γ and therefore drove recruitment of T cells that results in the clinical patterns characteristic of human vitiligo (71). The central role of the IFN-γ/chemokine axis in driving vitiligo, requiring activation of JAK1/JAK2, provided the rationale for highly successful treatment of the disease using JAK inhibitors, both orally and topically (Table 1) (69). Additional studies revealed that Tregs suppress melanocyte-reactive effector T cells (Teffs) in healthy individuals (72), are recruited to the skin by CCR6 (73), and their suppressive function in vivo required the activation of CCR5. This function is effective in nonlesional skin, but impaired or overwhelmed in lesional affected skin, likely through a disrupted Treg/Teff cell ratio (67).

The mechanism of melanocyte death in vitiligo is not well defined. Cytotoxic T cells utilize multiple pathways to kill their targets, including granzyme/perforin, FasL, cytokines, and others (74, 75). There are also multiple pathways that lead to cell death, including necrosis, apoptosis, necroptosis, pyroptosis, ferroptosis, autophagy, and others (76). An intriguing histological observation was that melanocytes within vitiligo lesions can be found at ectopic locations within the epidermis, above where they are normally located, which is anchored to the basement membrane in the basal epidermis (77). This suggests that melanocytes could be eliminated by the epidermis superficially during vitiligo progression, which is consistent with the lack of obvious melanocyte remnants in the dermis in most lesions. One study reported that this process could be initiated by the T cell–derived cytokines, IFN-γ and TNF-α, followed by production of matrix metalloproteinase 9 (MMP9) from keratinocytes that disrupts E-cadherin and causes melanocytes to detach from the basement membrane in reconstructed human pigmented epidermis (78). However, TNF-α inhibitors do not reverse vitiligo and even worsen the disease, bringing into question the role of this cytokine in disease pathogenesis (79–83). Further studies will be required to detail the mechanisms by which melanocytes in vitiligo are eliminated from the skin.

Relapse of depigmentation characterizes vitiligo in patients after discontinuing effective therapies, including conventional treatments like topical immunosuppressants, narrowband ultraviolet B (nbUVB), as well as JAK inhibitors (84, 85). This relapse first occurs at previously affected sites where treatment reversed disease through repigmentation. The source of this autoimmune “memory” of the skin was identified by multiple groups who reported the formation of autoreactive skin-resident memory CD8^+^ T cells (Trms) in both mouse models and human patients (86–90). These Trms act as sentinels that recognize attempted repigmentation of melanocyte stem cells and reinitiate autoimmune attack through the production of IFN-γ, induction of chemokine recruitment of Teffs from the blood, and destruction of the melanocyte stem cells (86). This activity is effectively inhibited by treatments; however, even JAK inhibition does not remove the cells from their niche (91), and thus Trms are responsible for both the maintenance and relapse of vitiligo.

Autoreactive Trms in vitiligo require IL-15 for their maintenance in the epidermis, and studies in mice revealed that blockade of IL-15 signaling removed the Trms from the skin, resulting in repigmentation even after a short course of treatment. Both mouse and human melanocyte-reactive T cells were found to express very high levels of the IL-15 receptor β chain (CD122), suggesting that these autoreactive cells, in contrast with nonautoreactive T cells that expressed lower levels of the receptor, were dependent on IL-15 signaling for their survival. This was supported by observations in the mouse model that CD122 antibody blockade also reduced the numbers of systemic autoreactive T cells in the blood and lymph nodes, without affecting nonautoreactive CD8^+^ memory T cells in these compartments (89). Together, these observations supported the hypothesis that “erasing” autoreactive memory from the skin through IL-15 blockade could result in long-term, durable treatment responses even after discontinuing therapy. This hypothesis is currently being tested in clinical trials.

Similar to vitiligo, CD8^+^ Trms have been identified in the target tissues of other autoimmune diseases, including patients with MS (92), T1D (93), and Crohn’s disease (94). An array of CD8^+^ T cell killing mechanisms, including perforin/granzyme and Fas/FasL, have been reported to mediate autoimmune destruction of melanocytes in vitiligo. In examinations of other autoimmune diseases, the granzyme/perforin pathway has been implicated in inflammatory bowel disease (IBD) (95), MS (96), Hashimoto’s thyroiditis (97), and T1D (98). With regard to FasL-mediated killing, autoreactive T cells from the NOD mouse model of T1D have been shown to utilize, but not require, this pathway for induction of T1D (99). Similarly, a CD8^+^ T cell–mediated mouse model of MS has also been reported to be exacerbated when the autoreactive CD8^+^ T cells express FasL (100). The use of multiple pathways by autoreactive CD8^+^ T cells to destroy target cells complicates achieving a mechanistic understanding of the killing process in autoimmune diseases. This is further complicated in diseases like T1D, MS, and IBD by the relative inaccessibility of the target tissue in humans for study. Because similar killing mechanisms are at play in vitiligo and the affected tissue is easily accessed, vitiligo is poised to offer key insights into CD8+ T cell–mediated autoimmune pathogenesis, including identifying the hierarchy of killing mechanisms used by autoreactive CD8^+^ T cells.

### Reversal of autoimmunity.

**Figure 3:**
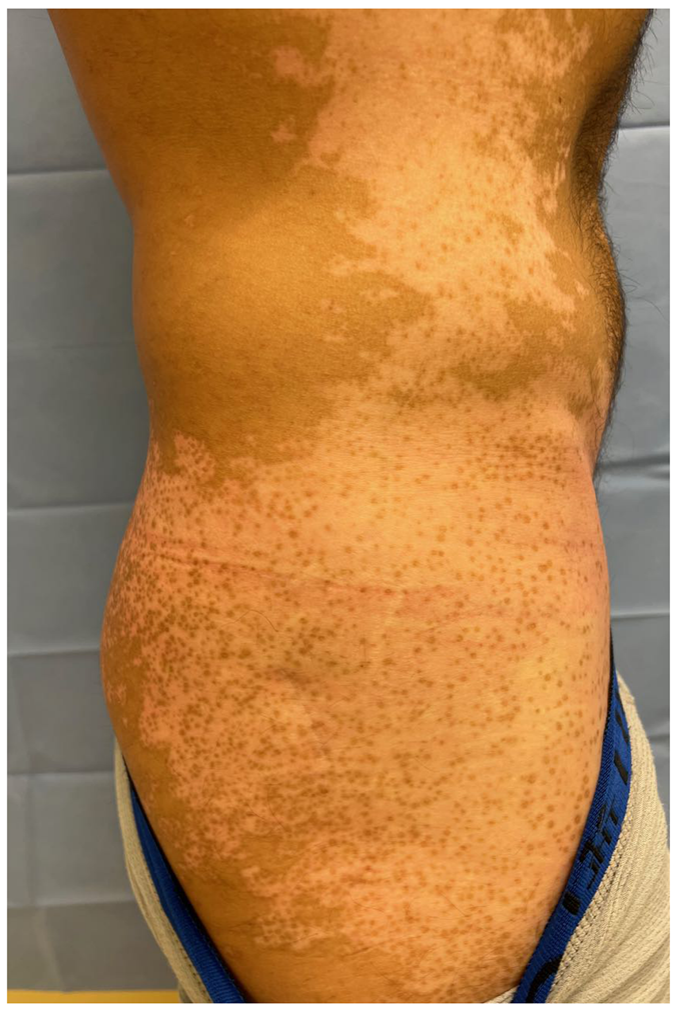
Perifollicular repigmentation of a large area with vitiligo. Pigmented spots are located around hair follicles. Photo is shown with patient consent.

Many autoimmune diseases are not reversible except by transplantation of the affected tissue/organ, a result of the absence of an appropriate stem cell population that can regenerate the target of autoimmunity. In contrast, vitiligo is reversible with treatment, via repigmentation of the skin through repopulation by melanocyte stem cells (101, 102). In normal skin, these cells reside in multiple locations, such as the epidermis, dermis, and appendages, including hair follicles and eccrine glands (103). However, while these cells appear to be destroyed in the epidermis by vitiligo autoimmunity, they are frequently spared in hair follicles, presumably due to immune privilege of this site, preventing the entry of autoreactive T cells into the melanocyte stem cell niche. The result is maintained pigmentation of hair follicles within patches of epidermal depigmentation (see Figure 1A) and successful repopulation of the epidermis by differentiated melanocytes that migrate out of each hair follicle, represented by the clinical observation of perifollicular repigmentation after treatment (102) (Figure 3). Consequently, areas of the skin without hair follicles (glabrous skin including the fingertips, bony prominences, volar wrists, dorsal feet, areola, mucosa, and penis), or areas where immune privilege has failed and the hair turned white, do not successfully repigment with therapy (104).

Optimal reversal of vitiligo would not only inhibit autoimmune attack of melanocytes, but also promote the proliferation, migration, differentiation, and function of melanocyte stem cells. One clinical study to address this used afamelanotide, an analog of α-melanocyte-stimulating hormone (α-MSH), but with a longer half-life. This accelerated the rate of repigmentation when receiving nbUVB, demonstrating the value of stimulating melanocyte regeneration; however, it resulted in significant darkening of the normal skin as well, thus enhancing the contrast and visibility of lesions (105, 106). Ongoing studies are testing this treatment in patients with darker skin to limit this contrast. Studies on repigmenting melanocytes revealed that multiple pathways, particularly β-catenin, were activated during regeneration (101, 107). Another study reported that cellular stress in melanocytes disrupts WNT signaling, while agonists of the WNT pathway induced differentiation of melanocyte stem cells into premelanocytes in vitiligo lesional skin, suggesting that this class of drugs may provide a novel approach to the treatment of vitiligo (108).

An important implication of the ability to reverse vitiligo after it appears is that the need to predict and prevent the condition is less important than other diseases. Furthermore, reversal can be achieved with topical (as opposed to systemic) therapy, reducing the risk of serious side effects that any immunosuppressive or immunomodulatory treatment carries with it. In contrast, when disease is not reversible, as in the case of T1D, treatments need to be started before disease manifests. This increases the importance of being able to identify at-risk individuals so that treatment is given only to those that are most likely to need it.

### Pathogenesis of segmental vitiligo.

Segmental vitiligo is characterized by focal depigmentation of the skin that does not typically cross the midline of the body (109) (see Figure 1, C and D). It frequently affects the hair follicles in the region early during evolution of the disease, and therefore is more difficult to treat, although it responds well to surgical transplantation of epidermal cells (110). The unilateral appearance of this variant previously evoked hypotheses on the neural origins of disease; however, there are currently no data to support this, and studies of early disease reveal T cell infiltration and autoimmunity that recapitulates the pathogenesis of vitiligo (6). The leading hypothesis is that melanocytes in the distribution of the disease are abnormal, possibly due to an acquired mosaic mutation in a melanocyte precursor during embryogenesis (111). Melanocyte precursors migrate ventrally from the dorsal neural crest until they meet in the midline, so the daughter cells of a precursor with a new mutation would also possess and propagate this change, creating a unilateral field of abnormal cells. If this abnormality promotes autoimmune inflammation, melanocyte destruction would follow and present as unilateral depigmentation that does not cross the midline.

## Treatment

The approach to treatment is influenced by various clinical factors, including the type, severity, and current activity level of vitiligo. The goal is to stop disease progression, stimulate and maintain repigmentation, as well as prevent disease relapse (112). In the last 10 years, guidelines for vitiligo treatment were published in several countries (109, 113–115), and recently a consensus statement was developed by the international Vitiligo Task Force (116). Both guidelines and the expert consensus statement recommend treatment with nbUVB phototherapy combined with systemic treatment for rapidly progressing vitiligo, such as oral steroid minipulse therapy, and topical treatment options such as corticosteroids or calcineurin inhibitors for stable and localized forms. Combination of topicals with phototherapy (full-body nbUVB units, excimer laser or lamp, and home phototherapy) are recommended to achieve optimal results. Biweekly application of 0.1% tacrolimus ointment as a topical immunosuppressant after achieving desired repigmentation was found to help maintain repigmentation with reduced risk of relapse (84). For patients with segmental vitiligo or other treatment-resistant, localized, and stable forms of vitiligo, surgical treatment that transfers melanocytes from unaffected skin to vitiligo lesions can be considered (117).

The identification of IFN-γ signaling as central to disease pathogenesis provided strong rationale to target this pathway therapeutically, leading to the development of topical ruxolitinib, the first FDA- and European Medicines Agency–approved treatment to reverse vitiligo, as well as four additional oral JAK inhibitors currently in clinical trials (118, 119). Combining ruxolitinib (JAK inhibitor) cream with nbUVB was well tolerated and resulted in improvement in facial and total body repigmentation (120). Real-world safety data suggest ruxolitinib cream is relatively safe, without significant systemic adverse events except a low incidence of application site reactions like irritation and acne (121).

**Figure 4:**
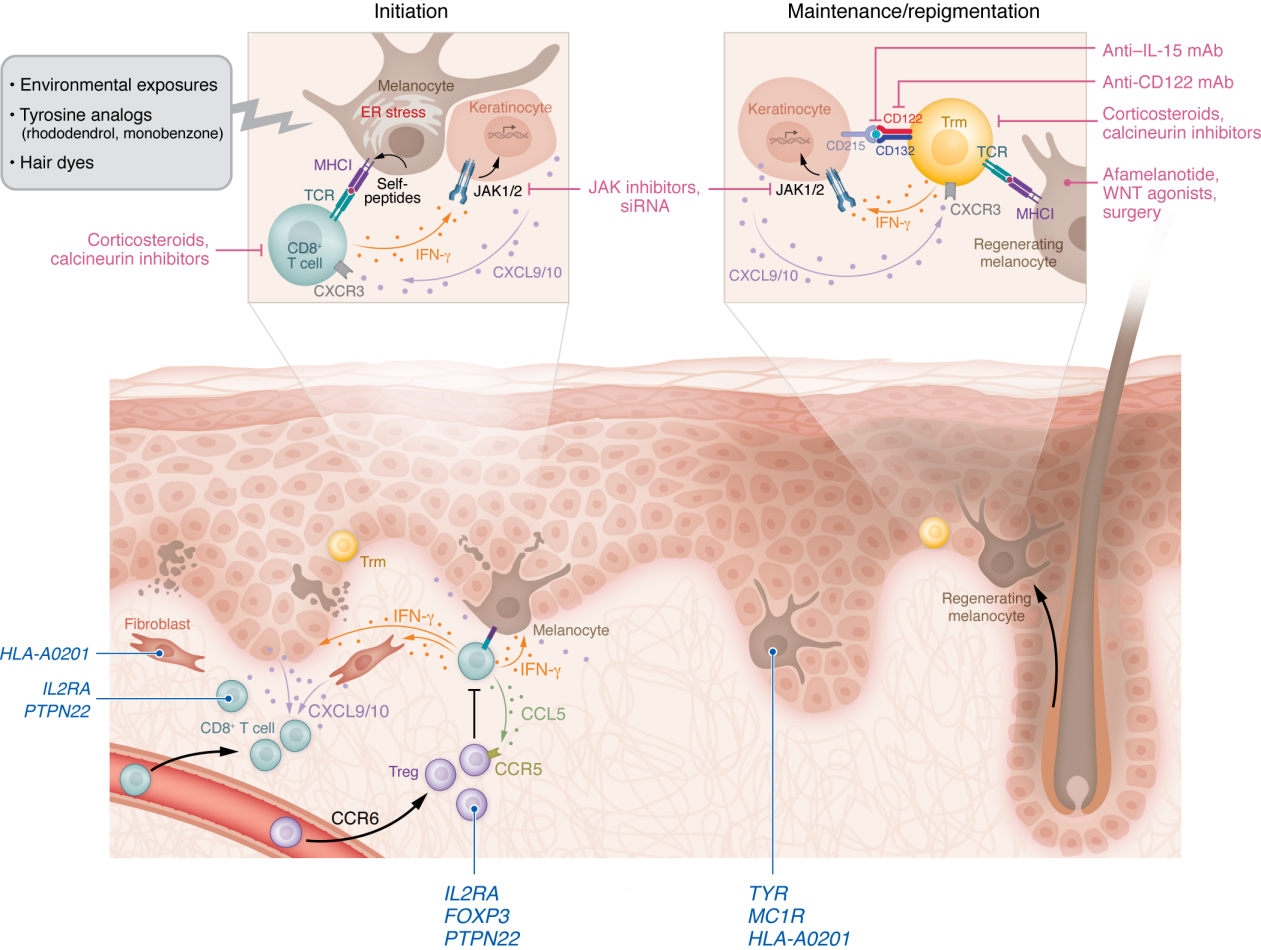
Mechanisms of vitiligo pathogenesis. Upper left: Initiation of pathogenesis within the vitiligo lesion is linked to intrinsic cellular stress and/or environmental exposures that induce release of damage-associated molecular patterns and antigen, which is acquired by phagocytic cells that travel to the draining lymph node (not shown) for initial priming of the T cell response. Tyrosine analogs such as rhododendrol and monobenzone (top left) are well-characterized examples of chemical exposures that induce autoimmunity. Lower left: Melanocyte-reactive CD8^+^ T cells enter the lesional skin and encounter their target melanocytes, promoting the release of IFN-γ, which induces production of CXCL9/10 from keratinocytes and fibroblasts that amplify the response by recruiting additional CD8^+^ T cells. Tregs, recruited to skin via CCR6, require CCR5 signaling for suppression of CD8^+^ T cell responses. Treg-mediated suppression is sufficient to block melanocyte-reactive T cell function in nonlesional skin, but is insufficient in lesional skin. Targets for therapeutic intervention are indicated in the upper panels (pink text), and candidate genetic variants from GWAS that influence initiation of the disease are indicated in the lower panels (dark blue text).

In randomized phase II clinical trials, oral ritlecitinib (a JAK3 and TEC family kinase inhibitor) and upadacitinib (a JAK1 inhibitor) were found to be effective and well tolerated in patients with active nonsegmental vitiligo over a period of 48–52 weeks (122, 123), supporting further investigation of effectiveness of these and other similar drugs in phase III studies. Other oral JAK inhibitors, baricitinib and povorcitinib (124), are also currently being tested in clinical trials, and these medications should expand the therapeutic options available in the coming years. Ongoing clinical trials for vitiligo are listed in Table 1. Novel treatment approaches continue to target the IFN-γ pathway in vitiligo, including an epidermally tethered antibody to neutralize IFN-γ (125), as well as siRNAs that target the IFN-γ receptor (IFNGR1) and JAK1 (126–128). Delivery of JAK1 siRNA targeted to the skin to disrupt IFN-γ signaling at the treatment site reduced T cell infiltration and prevented depigmentation in a mouse model (127). Understanding vitiligo on a genetic and molecular level (Figure 4) offers several potential future therapeutic alternatives, such as treatments targeting IL-15, HSP70i, MMP9, or WNT signaling, as discussed above (35, 78, 89, 108, 129, 130). It is not surprising that many of these potential vitiligo treatments are also being evaluated in other autoimmune diseases (Table 1). Of course, as a skin disease, vitiligo is treatable with topical therapies, which will not be relevant for diseases of internal organs. Thus, translating new vitiligo treatments to other diseases will require systemic administration and consideration of the associated risks.

## Skin cancer and other diseases

There is an inverse relationship between susceptibility to vitiligo and skin cancer risk for both melanoma and non-melanoma skin cancer, which appears to be related to genetic factors (26). This determination was initially made from a GWAS indicating that the rs1126809 variant of the tyrosinase gene *TYR*, which is involved in converting tyrosine to melanin, increased the risk of vitiligo and reduced the risk of melanoma (131). More recently, another GWAS investigated the role of 58 genetic loci associated with vitiligo in skin cancer risk. The combined analysis of two melanoma studies identified four vitiligo susceptibility loci, including RALY-EIF252-ASIP-AHCY-ITCH, IRF4, TYR, and MC1R. All four of these melanoma-associated loci were also significantly associated with the risk of basal cell (BCC) and squamous cell (SCC) carcinomas. Within the 58 loci examined, all showed an inverse correlation between the risk of vitiligo and both melanoma/SCC, while 80% showed an inverse correlation for BCC (132).

Over the past decade, several large epidemiological studies confirmed the inverse association between vitiligo and new-onset skin cancer (133–135), while in patients with stages III and IV melanoma treated with immunotherapy, onset of vitiligo-like depigmentation is significantly associated with prolonged disease-free survival as well as overall survival (57). In addition, the use of topical calcineurin inhibitors, phototherapy, or both treatments in combination did not increase the risk of cancers (136–140). In fact, patients with vitiligo experienced a significantly reduced risk of overall internal malignancies compared with control individuals without vitiligo (141), and even demonstrated a lower incidence of all-cause mortality, including infections, cancer deaths, and cardiovascular disease, indicating a protective effect of having the disease (142). These observations indicate that those with vitiligo may possess enhanced immunity that translates to increased protection from other diseases, an association coined the “white armor” (143, 144). To date, clinical trials have not revealed cancer risks in emerging immunosuppressive treatments such as JAK inhibition or melanocyte-activating treatments like afamelanotide, although it may require longer observation periods to definitively rule out these risks.

Consistent with enhanced immunity, numerous studies have established a correlation between vitiligo and other autoimmune diseases within the patient or family members, including thyroid disorders, alopecia areata, rheumatoid arthritis, T1D, Addison’s disease, and pernicious anemia (27). Given the heightened susceptibility to autoimmune thyroid disease in vitiligo, most notably Hashimoto’s thyroiditis (145), an initial screen for thyroid-stimulating hormone levels is commonly performed for patients with new-onset vitiligo.

## Conclusions

As noted throughout this Review, vitiligo shares mechanisms of autoimmunity with many other similar autoimmune diseases. These diseases clinically present in a similar way, with ongoing target cell destruction going unnoticed by the patient (largely asymptomatic), with symptoms appearing only after the loss of target cell function, such as pigment loss in vitiligo, insulin loss leading to ketoacidosis in T1D, loss of thyroid hormone and associated symptoms in Hashimoto’s thyroiditis, lack of intrinsic factor for B12 absorption in pernicious anemia, and loss of adrenal hormones leading to disrupted homeostasis in Addison’s disease. This asymptomatic autoimmunity is in contrast with diseases that directly cause symptoms such as itch or pain from inflammation, including atopic dermatitis, psoriasis, rheumatoid arthritis, IBD, etc. To highlight these similarities and distinguish this group of diseases from tissue-specific, but not cell-specfic, diseases like lupus, rheumatoid arthritis, or psoriasis, we propose the term “cell-specific autoimmune diseases” for this group.

The relatedness among these diseases is further supported by the prevalence of polyautoimmunity, including overlap of two or more of these diseases in individual patients, increased prevalence of these diseases in close family members, and overlap of genetic risk alleles among this family of diseases. Most importantly, disease mechanisms are shared across these diseases, including dependence on T cell–mediated cytotoxicity, IFN-γ inducing type 1 inflammation, cellular stress caused by the unfolded protein response and reactive oxygen species, as well as environmental exposures that increase the risk of disease (40). Thus, vitiligo is one member of a family of cell-specific autoimmune diseases that affect diverse organ systems.

Vitiligo is an excellent model for understanding related cell-specific autoimmune diseases and tumor immunity that are more difficult to study. The disease is highly prevalent (~1%); the affected skin can be clinically observed and readily sampled; the target cell and many of its antigens are known; environmental factors that induce the disease are characterized; translational research tools are readily available; spontaneous regeneration of targeted cells readily occurs, making disease reversal possible without transplant or stem cell application; and clinical trials are efficient, leading to rapid proof of concept for novel therapeutics (6). Thus, vitiligo provides an unparalleled model through which to understand mechanisms of both tumor immunotherapy and cell-specific autoimmunity. When novel treatments are developed and proven effective for vitiligo, these can be repurposed for related diseases, eliminating the difficult, time-consuming, and costly need to “reinvent the wheel” for each disease.

## Figures and Tables

**Table 1:**
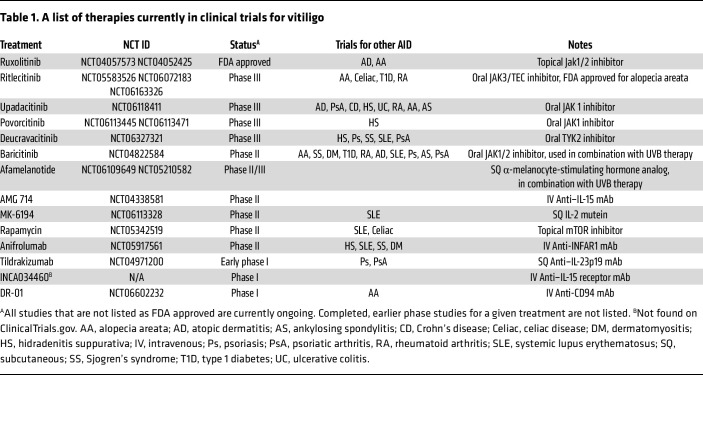
A list of therapies currently in clinical trials for vitiligo

## Version 1 01/16/2025

Electronic publication
